# Cavernous Hemangioma-Like Kaposi Sarcoma: Histomorphologic Features and Differential Diagnosis

**DOI:** 10.1155/2013/959812

**Published:** 2013-09-25

**Authors:** Nilüfer Onak Kandemir, Figen Barut, Banu Doğan Gün, Nilgün Solak Tekin, Sevinç Hallaç Keser, Şükrü Oğuz Özdamar

**Affiliations:** ^1^Department of Pathology, Faculty of Medicine, Bülent Ecevit University, 67100 Zonguldak, Turkey; ^2^Department of Dermatology, Faculty of Medicine, Bülent Ecevit University, 67100 Zonguldak, Turkey; ^3^Department of Pathology, Dr. Lütfi Kırdar Training and Research Hospital, Istanbul, Turkey

## Abstract

*Aim*. Cavernous hemangioma-like Kaposi sarcoma is a rare morphologic type of Kaposi sarcoma. So far there are no cases in the literature defining the histological features of this morphologic spectrum in detail. In this study we presented two classical-type cutaneous Kaposi sarcoma cases with histologic findings resembling cavernous hemangioma in company with clinical and histopathological data. *Cases*. One hundred and eighty-five classical-type cutaneous Kaposi sarcoma lesions in 79 patients were assessed retrospectively in terms of histopathological features. Findings of two cases showing features of cavernous hemangioma-like Kaposi sarcoma whose clinical data could be accessed were presented in accompany with the literature data. Both cases were detected to have bluish-purple, protruded, irregularly bordered cutaneous lesions. Histopathological examination revealed a lesion formed by cavernous hemangioma-like vascular structures organized in a lobular pattern that became dilated and filled with blood. Typical histological findings of early-stage KS, consisting of mononuclear inflammation, extravasated erythrocytes, and a few immature vascular structures in superficial dermis, were observed. All cases were serologically HIV-1 negative. A positive reaction with HHV-8, CD31, CD34, and D2-40 monoclonal antibodies was identified at both cavernous hemangioma-like areas and in immature vascular structures. *Results*. Cavernous hemangioma-like Kaposi sarcoma is a rare Kaposi sarcoma variant presenting with diagnostic challenges, that may be confused with hemangioma. As characteristic morphological features may not be observed in every case, it is important for diagnostic purposes to show immunohistochemical HHV-8 positivity in this variant.

## 1. Introduction

Kaposi sarcoma (KS) is a rare vascular lesion with four different epidemiological forms (classical, African (endemic), iatrogenic, and AIDS related) caused by human herpes virus-8 (HHV-8) [1]. It may have various clinical and histopathological features. More than 10 histological subtypes has been defined so far (e.g., anaplastic, lymphangioma-like, and micronodular KS) [2, 3]. While clinical appearance of lesions is different from usual type KS in some subtypes (e.g., bullous, verrucous, and ecchymotic KS), most histological subtypes do not have a prominent discriminatory clinical feature [1–7]. Despite being variable by lesion stage, some characteristic findings are important in histopathological diagnosis. Among these are immature vascular formations intersecting collagen to form cleft- or mesh-like patterns and spindle cells that are considered to be the main neoplastic component. Hyaline globules, hemosiderin pigment, and lymphoplasmacytic inflammatory cells frequently accompany neoplastic vascular structures [1, 8]. In KS spindle cells and neoplastic vascular structures react positively with endothelial markers (e.g., CD31, CD34, and D2-40) [1]. The most important point in the diagnosis of KS is to show HHV-8 immunopositivity supporting histological findings in the neoplastic component [9–11].

Since they are considerably rare, we discussed two cases of classical-type cutaneous KS exhibiting cavernous hemangioma-like features in company with clinical and histopathological findings and stressed the importance of HHV-8 immunoreactivity in differential diagnosis of these lesions.

## 2. Case Report

This study enrolled 185 cases of classical-type cutaneous KS lesions of 79 patients who were diagnosed at Bülent Ecevit University, Faculty of Medicine, Department of Medical Pathology, and Dr. Lütfi Kırdar Kartal Training and Research Hospital between 2001 and 2012 and whose data could be retrieved. Clinical information of the cases was obtained by screening medical files and reassessing materials of pathology archive for histopathological and immunohistochemical data. No patient had a history of immunosuppressive drug use or transplantation. All patients were human immunodeficiency virus-1 (HIV-1) negative. 

### 2.1. Histopathological and Immunohistochemical Assessment

Diagnosis of KS was confirmed by reviewing of Hematoxyline&Eosin (H&E)-stained sections. Histopathological progression of the lesions were grouped into early (patchy), intermediate (plaque), and late (nodule/tumor)-stage KS according to previously specified criteria [1, 2, 8]. All lesions were examined immunohistochemically with HHV-8 (LNA-1) and endothelial markers (CD31, CD34, and D2-40).

Among retrospectively examined biopsy samples, lesions, the dominant histopathological component which is composed by cavernous hemangioma-like vascular structures and in which typical histological features of KS are observed at focal areas, were classified as cavernous hemangioma-like Kaposi sarcoma (CHLKS) (see Figure 2). Vascular structures that were lined by flattened endothelium, dilated, and filled by blood were considered to have cavernous hemangioma-like feature. Characteristic findings of KS (e.g., those of immature vascular proliferations that are infiltrative bordered, contain intracytoplasmic lumen, and form mesh- and cleft-like structures and/or spindle cell proliferation) were assessed in accordance with the previously specified criteria [1, 2, 8].

## 3. Results

Retrospective assessment of archive materials of 185 classical-type cutaneous KS lesions belonging to 79 patients revealed that histopathological features of lesions of 2 cases were consistent with CHLKS. Clinical, histopathological, and immunohistochemical findings of these cases were summarized below.

### 3.1. Case 1

#### 3.1.1. Clinical Assessment

A 69-year-old man presented with purple spots at both feet. The lesions had begun at the lateral aspects of the feet and their number and size increased gradually. He had a 10-year hypertension history for which he had been taking medications. Dermatologic examination revealed multiple purple-colored plaques and papules of 0.5–1.5 cm, some of which were covered with crust on the dorsum of feet and around the ankles. He had no pathology in laboratory and serologic examinations. Physical examination revealed no signs suggestive of systemic involvement of KS, including suggestive symptoms, lymphadenopathy, and visceral involvement. He was HIV-1 negative in serological examination. Biopsies were taken from the lesions with an initial diagnosis of KS. 

The patient underwent cryotherapy for the Kaposi sarcoma lesions in lower extremity. The patient was followed at 6-month intervals for 22 months, and new cutaneous lesions of Kaposi sarcoma in lower extremities were treated with cryotherapy. No systemic involvement or an aggressive course was present.

#### 3.1.2. Histopathological Findings

H&E sections of the lesion demonstrated a dilated, blood-filled vascular proliferation lined by flattened endothelium stretching from papillary dermis under hyperkeratotic squamous epithelium to reticular dermis, forming lobular patterns. A few thick-walled vascular structures were observed in that lesion that was composed of thin-walled vessels. A hemosiderin accumulation was noted at the vessel wall and surrounding stroma. Chronic inflammatory cells and histological findings of early-stage KS such as vascular structures forming clefts, hemosiderin pigment, and extravasated erythrocytes were observed around the cavernous hemangioma-like vascular structures, and these findings were more prominent at papillary dermis. At these areas a prominence of nucleolus and mild atypia was notable in endothelial cells lining vascular structures. The lesion did not show spindle cell proliferation, a finding observed at advanced stages of KS. Cavernous hemangioma-like vascular structures formed about 80% of the lesion, and typical histopathological findings of early-stage KS were observable at focal sites inside the lesion.

### 3.2. Case 2

#### 3.2.1. Clinical Assessment

A 70-year-old female patient presented with the complaint of purple discolorations on the dorsum of the right foot, which had been present for 2 years and growing in number. Her past history was remarkable for an operation against colon carcinoma 10 years ago. She had not taken chemotherapy and radiotherapy. She also had goiter for 5 years. Dermatologic examination revealed multiple purple-colored macular lesions with diameters ranging from 0.5 to 1 cm on the dorsum of the right foot. Her serologic examination was negative for HIV-1. Systemic examination and laboratory tests were negative for a finding suggestive of systemic involvement of KS. Biopsies were taken from the lesions with an initial diagnosis of KS. 

The patient was treated with local excision and cryotherapy for cutaneous Kaposi sarcoma lesions. The patient was followed at six month periods for a total of 11 months. During follow-up, local recurrences were detected in lower and upper extremities and treated with cryotherapy. No systemic involvement or an aggressive course was present during follow-up.

#### 3.2.2. Histopathological Findings

H&E sections of the lesion revealed a vascular proliferation forming more than 90% of the lesion at the dermis, which had similar features with the first case. The lesion was composed of vascular structures filled by blood, which were lined by flattened endothelium and organized as lobular patterns, some of which included fibrous septa. Some vascular structures were closely spaced from skin adnexae that were surrounded by hemosiderin pigment. At the papillary dermis adjacent to the lesion were immature vascular structures intersecting collagen and extravasated erythrocytes, findings of the early-stage KS. No spindle cell proliferation was evident in the lesion.

#### 3.2.3. Immunohistochemical Findings

Immunohistochemical examinations of lesions from both cases showed a strong reaction with endothelial markers (CD31, CD34, and D2-40) and HHV-8 (LNA-1) both in endothelia of the cavernous hemangioma-like vascular structures and at the areas of early-stage KS. Endothelia of the mature vascular structures adjacent to the lesions had a positive reaction with Factor VIIIra whereas neoplastic vascular structures were negative. A focal positive reaction was present with actin and desmin at the walls of the cavernous hemangioma-like vascular structures. In the light of clinical, histopathological, and immunohistochemical findings, both lesions were interpreted as classical-type early-stage cutaneous KS, that showed features similar to cavernous hemangioma.

## 4. Discussion

KS is a vascular neoplasm with a low malignancy potential, which is caused by HHV-8, has four different epidemiological forms, and has considerably different clinical and histopathological features [1, 8, 12]. Cases in which the typical histological features of KS are dominant are designated as usual type KS, and these lesions are classified into patch, plaque, and nodule phases, depending on the clinical progression [1]. The most common histological subtype in all epidemiological forms of KS is the usual type KS [2, 3]. Other common histological subtypes include lymphangioma-like and lymphangiectatic types [2–4, 13]. While bullous, verrucous, and ecchymotic type KS lesions include clinically distinguishable features, most histological subtypes cannot be distinguished clinically from the usual type KS lesions [2, 5, 6]. Anaplastic KS and lymphangioma-like KS have been reported to have a more aggressive course than usual type KS [2, 4, 14]. 

A wide spectrum of clinical and histopathological morphologies of KS poses a difficulty in differential diagnosis and a delay in the diagnosis and treatment of the disease [15, 16]. In certain areas of our country classical-type KS is a commonly encountered cutaneous vascular neoplasm. Thus, knowledge of the histological subtypes and a search for the presence of the virus HHV-8 in cases suspected for KS are important for differential diagnosis [9–11].

Studies investigating histological subtypes of KS have been mostly case reports including a low number of cases. There are a few studies studying histological subtypes of KS in large samples [2–6, 16, 17]. In this study 185 classical-type cutaneous KS lesions from 79 patients were retrospectively assessed, and two cases showing cavernous hemangioma-like features were reported with regard to histopathological features and differential diagnosis in company with the literature data. 

In our study both cases considered as having CHLKS were elderly patients having multiple cutaneous lesions of blue-purple color located in lower extremity. These lesions include typical clinical findings of early-stage KS and do not contain distinguishing features. No history of drug use with attending immunosuppression, transplantation, or HIV infection was notable after clinical and laboratory examinations in the patients.

The common histopathological features of the lesions considered as CHLKS were formation of nearly all lesions by cavernous hemangioma-like vascular structures and the presence of typical histological features of early-stage KS at focal areas. The congested vascular structures forming the main component of the lesions are lined by flattened endothelium, and an intimal thickening is notable at the walls of some vascular structures. Although there are no evident atypia in endothelial layer, some endothelial cells became prominent to form protrusions into the lumen. Hemosiderin pigment accumulation is observed at the vessel walls and the surrounding stroma (see Figure 4). In the first case, cavernous hemangioma-like vascular structures formed nearly the entire lesion, and typical areas of early-stage KS were hardly distinguished. In the second case, on the other hand, characteristic findings of early-stage KS including chronic-type inflammatory cells at the papillary dermis adjacent to the lesion were more marked. It was noteworthy in one case that cavernous hemangioma-like vascular structures were in close proximity with skin adnexae without infiltrating them (see Figure 3). Immunohistochemical examination of lesions of both cases revealed a strong positive reaction with endothelial markers (CD31, CD34, and D2-40) and HHV-8 (LNA-1) in both endothelial lining of cavernous hemangioma-like vascular structures and at areas of typical early-stage KS. While a positive reaction with Factor VIIIra was observed in endothelia of the mature vascular structures neighboring the lesion, no reaction was observed in neoplastic vascular structures. A focal positive reaction with actin and desmin was detected at the walls of the cavernous hemangioma-like vascular structures. In the light of all these clinical, laboratory, and histopathological findings, these two cases were interpreted as classical-type cutaneous KS showing features similar to cavernous hemangioma. 

**Figure 1:**
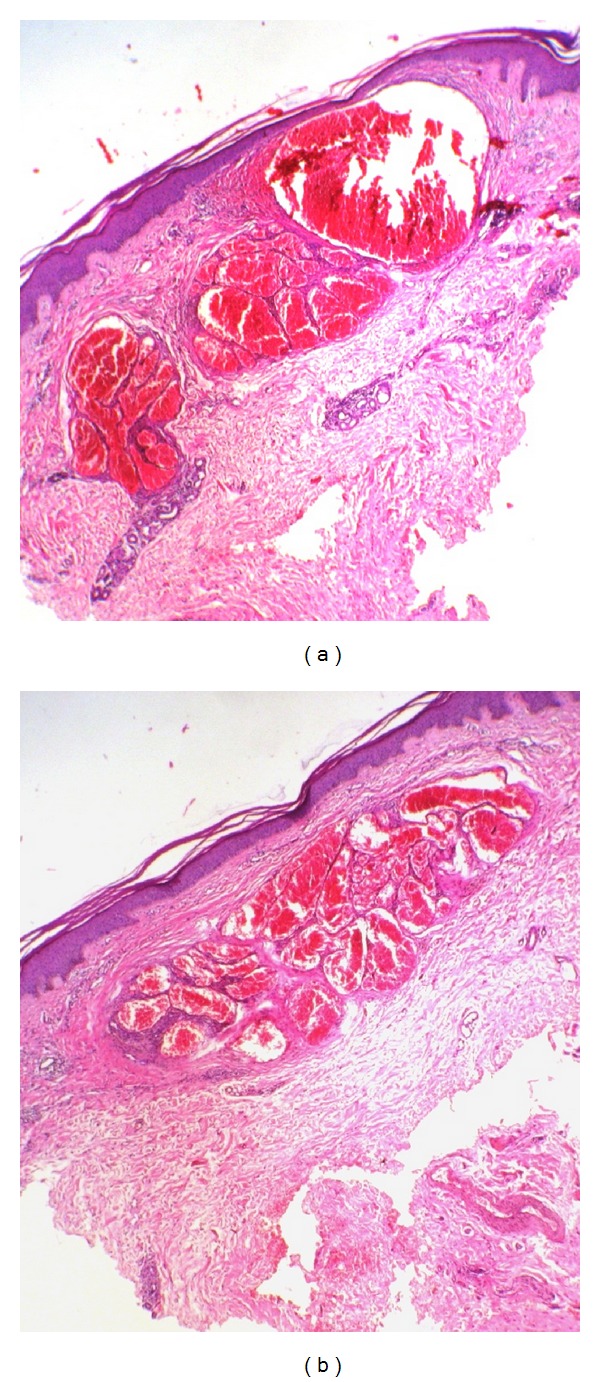
Biopsy samples of both cases reveal lesions composed of dilated vascular structures with blood-filled lumen. (a)-(b) Hematoxylin and Eosin (×40).

**Figure 2:**
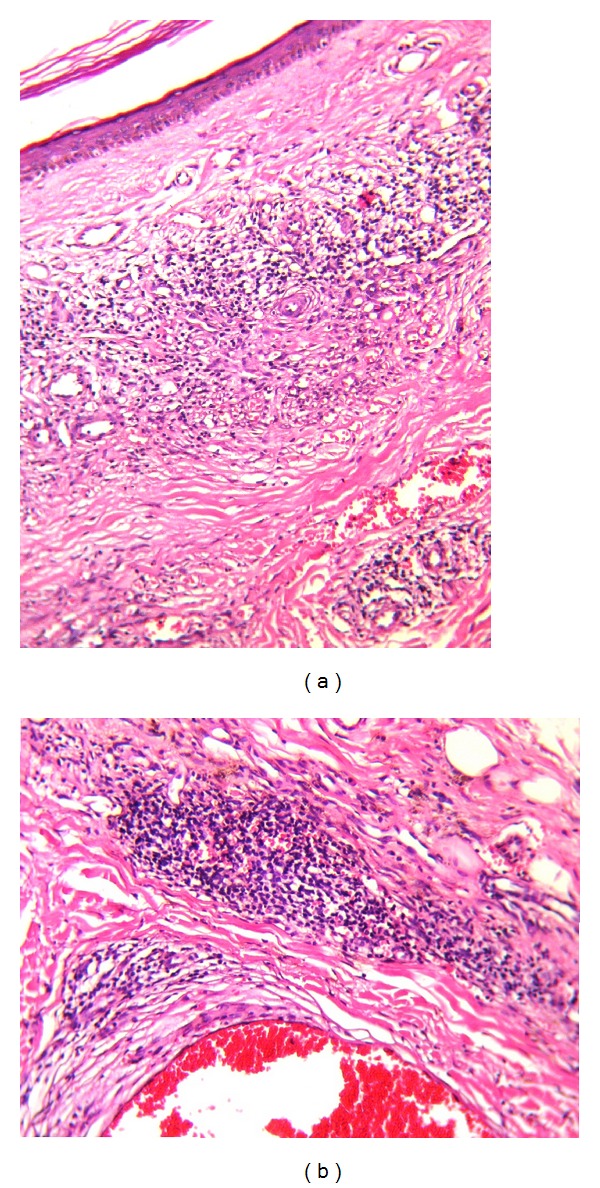
Microphotographs of both cases show the characteristic findings of early-stage Kaposi sarcoma surrounded by mononuclear inflammatory cells adjacent to cavernous hemangioma-like vascular structures. (a)-(b) Hematoxylin and Eosin ((a) ×40; (b) ×100).

**Figure 3:**
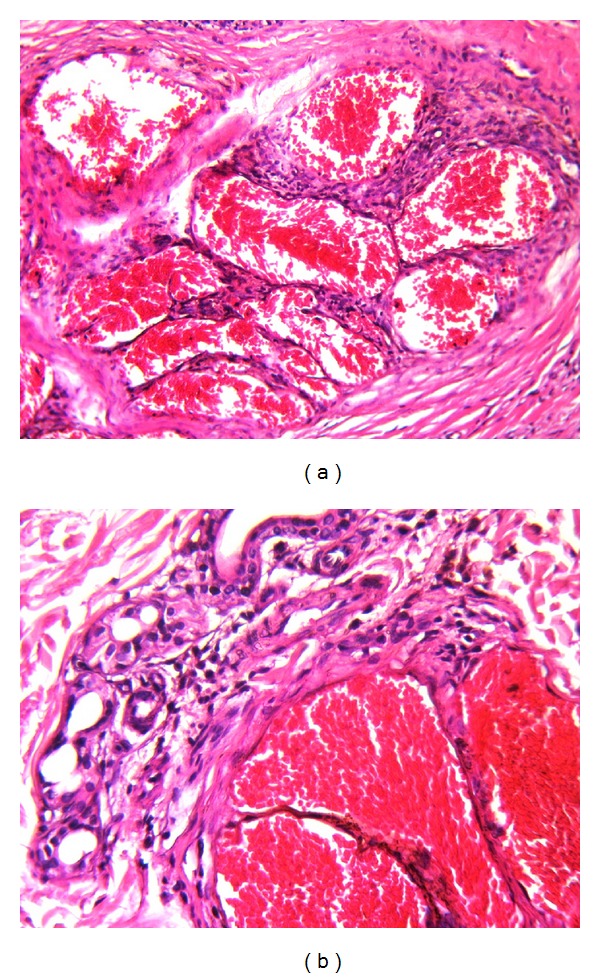
(a) Vascular structures with a thick wall are relatively well demarcated from the surrounding dermis. (b) Vascular proliferation is in close neighborhood to skin adnexae. (a)-(b) Hematoxylin and Eosin (×100).

**Figure 4:**
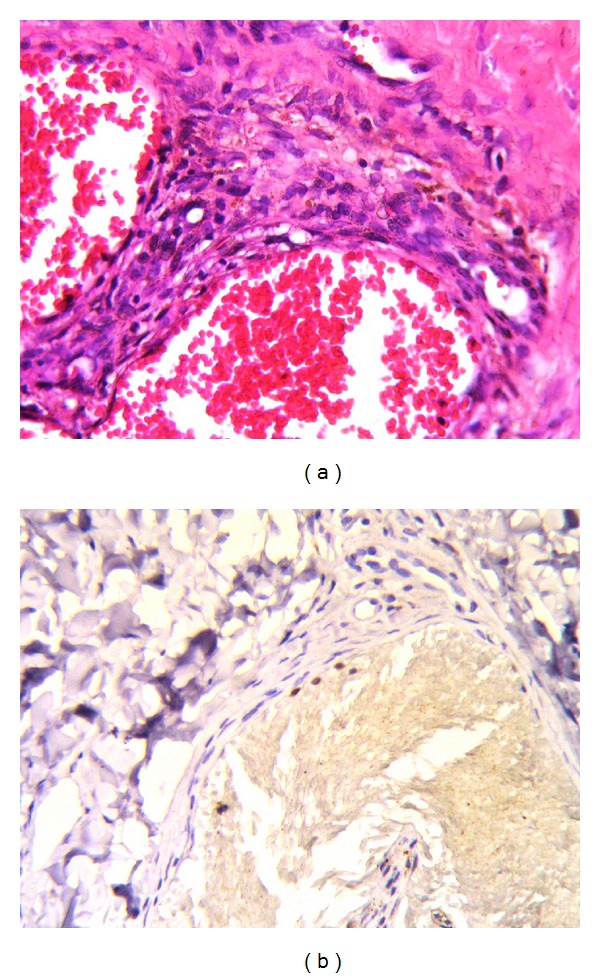
(a) Hemosiderin pigment is seen both on the walls of cavernous hemangioma-like vascular structures and in the stroma. (b) A positive immune reaction with HHV-8 is evident in endothelial cells. (a) Hematoxylin and Eosin (×100), (b) LSAB-DAB (×100).

**Figure 5:**
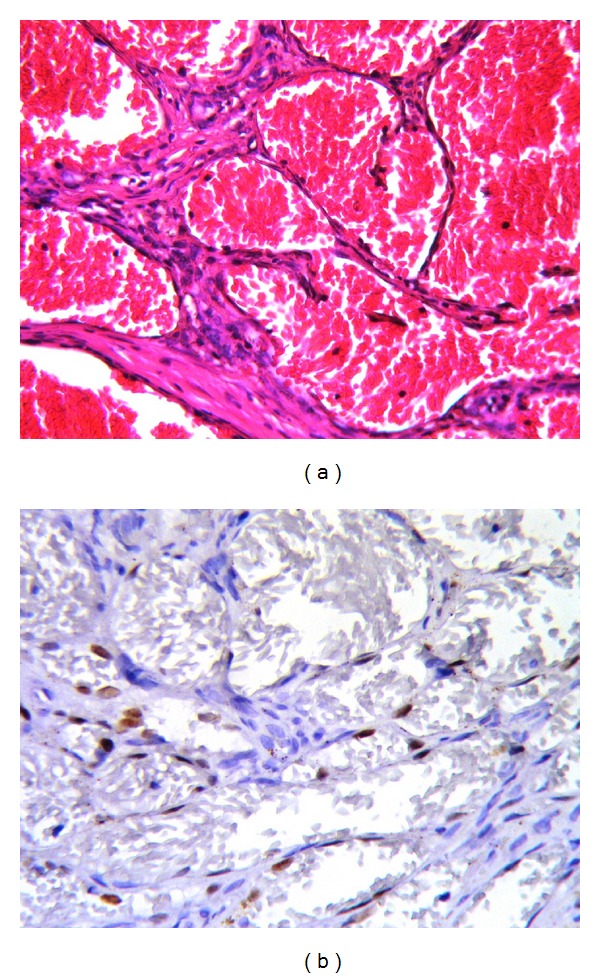
The anastomosing vascular structures contain flattened endothelial layer giving a positive immune reaction with HHV-8. (a) Hematoxylin and Eosin (×100), (b) LSAB-DAB (×100).

Differential diagnosis of CHLKS includes many reactive and benign vascular lesions [1, 15, 16]. In addition, dilated vascular structures resembling cavernous hemangioma may also be seen around the spindle cell proliferation in some lesions at the nodular stage of the usual type of KS (see Figure 1). These vascular formations do not contain a prominent wall, and they consist of either spindle cells giving a positive reaction with HHV-8 or a single-row endothelial layer (see Figure 5). In these lesions spindle cell proliferation typical of advanced-stage KS is prominent [1, 2, 18].

The most important lesion in the differential diagnosis of CHLKS is cavernous hemangioma. Cavernous hemangioma is a vascular lesion that is seen particularly in childhood period and more commonly involves the upper part of the trunk [1, 19, 20]. These lesions have a less defined border and a deeper location, and they may contain degenerative changes including thrombus and dystrophic calcification. Cavernous hemangiomas have dilated vascular structures lined by flattened endothelium, some of which have thick walls containing intimal fibrosis. Mature endothelial cells in cavernous hemangioma give a positive reaction with Factor VIIIra, and pericytic cells give a positive reaction with actin/desmin. Histological differential diagnosis from KS importantly includes spindle cell proliferation, endothelial atypia, and inability to see neoplastic and abnormal vessels intersecting collagen. Absence of immunohistochemical HHV-8 reaction is a finding in favor of cavernous hemangioma [1, 15, 19, 20].

Another lesion in the differential diagnosis of CHLKS is spindle cell hemangioma. Clinically, they present with purple-red, painless, nodular at the acral regions. Histologically, they are formed by spindle cell proliferation forming clefts intertwined with thin-walled cavernous hemangioma-like vascular structures. In some cases, similar to hemangioendotheliomas, an intracytoplasmic lumen or a vacuole may be seen in neoplastic cells. The component consisting of spindle cells does not react with endothelial cell markers and it is positive for vimentin/actin/desmin. Differential diagnosis with KS is possible with histological findings as well as immunohistochemical features supporting the pericytic origin of the spindle cells and an absence of HHV-8 positivity [1, 15, 19–22]. 

Presence of cavernous hemangioma-like features in only 2 (1%) cases of 185 lesions present in our study suggests that this histological finding is a rare morphological spectrum of KS. Macroscopic appearance of the lesions and the clinical data of the cases are consistent with classical KS. Whether this different morphological appearance may be considered as a histological subtype of KS will be elucidated by future studies with larger sample size.

## 5. Conclusion

CHLKS has a histological appearance very closely resembling a hemangioma, in which typical morphological findings of KS are not prominent. Thus, a careful morphological examination as well as demonstration of HHV-8 immunoreactivity is important for the diagnosis.
